# Optimization and validation of a virus‐like particle pseudotyped virus neutralization assay for SARS‐CoV‐2

**DOI:** 10.1002/mco2.615

**Published:** 2024-06-14

**Authors:** Shuo Liu, Li Zhang, Wangjun Fu, Ziteng Liang, Yuanling Yu, Tao Li, Jincheng Tong, Fan Liu, Jianhui Nie, Qiong Lu, Shuaiyao Lu, Weijin Huang, Youchun Wang

**Affiliations:** ^1^ Changping Laboratory Beijing China; ^2^ Chinese Academy of Medical Sciences & Peking Union Medical College Beijing China; ^3^ Division of HIV/AIDS and Sex‐Transmitted Virus Vaccines National Institutes for Food and Drug Control (NIFDC) Beijing China; ^4^ CAS Key Laboratory of Infection and Immunity, National Laboratory of Macromolecules, Institute of Biophysics Chinese Academy of Sciences Beijing China; ^5^ University of Chinese Academy of Sciences Beijing China; ^6^ National Kunming High‐level Biosafety Primate Research Center, Institute of Medical Biology, Chinese Academy of Medical Sciences and Peking Union Medical College, Kunming Yunnan, China Kunming China

**Keywords:** neutralizing antibody, pseudotyped virus, SARS‐CoV‐2, virus‐like particle (VLP)

## Abstract

Spike‐protein‐based pseudotyped viruses were used to evaluate vaccines during the COVID‐19 pandemic. However, they cannot be used to evaluate the envelope (E), membrane (M), and nucleocapsid (N) proteins. The first generation of virus‐like particle (VLP) pseudotyped viruses contains these four structural proteins, but their titers for wild‐type severe acute respiratory syndrome coronavirus 2 (SARS‐CoV‐2) are relatively low, even lower for the omicron variant, rendering them unsuitable for neutralizing antibody detection. By optimizing the spike glycoprotein signal peptide, substituting the complexed M and E proteins with SARS‐COV‐1, optimizing the N protein with specific mutations (P199L, S202R, and R203M), and truncating the packaging signal, PS9, we increased the titer of the wild‐type VLP pseudotyped virus over 100‐fold, and successfully packaged the omicron VLP pseudotyped virus. The SARS‐CoV‐2 VLP pseudotyped viruses maintained stable titers, even through 10 freeze–thaw cycles. The key neutralization assay parameters were optimized, including cell type, cell number, and viral inoculum. The assay demonstrated minimal variation in both intra‐ and interassay results, at 11.5% and 11.1%, respectively. The correlation between the VLP pseudotyped virus and the authentic virus was strong (*r* = 0.9). Suitable for high‐throughput detection of various mutant strains in clinical serum. In summary, we have developed a reliable neutralization assay for SARS‐CoV‐2 based on VLP pseudotyped virus.

## Graphical abstract

Through the optimization of the structural proteins of coronavirus virus‐like particle (VLP) pseudotyped virus, we successfully constructed high titer the VLP pseudotyped virus variant. We optimized the conditions for neutralizing antibodies and validated the methodology. We have established a detection method for neutralizing antibodies against severe acute respiratory syndrome coronavirus 2 (SARS‐CoV‐2) VLP pseudotyped virus, which shows good consistency with authentic viruses and suitable for detecting various variants.

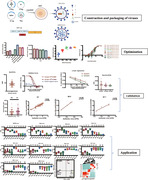

## 1 INTRODUCTION

In December 2019, an unprecedented strain of coronavirus (2019‐nCoV) was identified in novel instances of viral pneumonia emerging in Wuhan.^1^, ^2^, ^3^ COVID‐19, a highly contagious illness that has led to globally devastation, was officially classified as a pandemic by the World Health Organization (WHO) on March 11, 2020.^4^ 2019‐nCoV is the seventh coronavirus capable of infecting humans. Among the six other types of coronaviruses that impact humans are low‐pathogenic viruses: HCoV‐OC43, HCoV‐HKU1, HCoV‐NL63, and HCoV‐229E, and highly pathogenic severe acute respiratory syndrome coronavirus 1 (SARS‐CoV‐1) and MERS‐CoV.^5^, ^6^ Genetic comparison has revealed that the virus closely related to SARS‐CoV‐2 is the bat SARS‐like coronavirus RaTG13, which shares 96.2% sequence similarity.^6^ SARS‐CoV‐2 is characterized by a large, positive‐sense, single‐stranded genomic RNA that approximately 30 kb in length. This RNA encodes 16 nonstructural proteins and four structural proteins: the membrane (M), envelope (E), spike (S), and nucleocapsid (N) proteins.^7^ Spike of the virus directly interacts with the ACE2 protein and is the main target of vaccines and therapeutic drugs.^8^, ^9^ Spike protein enables the virus to attach and penetrate the target cell, thereby initiating the infection. Additionally, the S protein plays a crucial role in the induction of the protective immune responses during SARS‐CoV‐2 infection, making it a prime focus for the development of vaccines and therapeutic methods.^10^, ^11^, ^12^

Neutralizing antibodies, especially which elicited through vaccination or natural infection, are essential in preventing and defending against infectious diseases. Currently, various antiviral drugs have already been used to against SARS‐CoV‐2 infections, such as paxlovid and molnupiravir.^13^, ^14^, ^15^ Many vaccines have also shown promising results in clinical trials and have been granted conditional market authorization.^16^, ^17^, ^18^, ^19^ Nevertheless, it is still crucial to assess the efficacy of these vaccines regularly, in the capabilities of vaccine‐derived neutralizing antibodies against new viral variants.

The use of an authentic virus in research requires a biosafety level 3 facility and adherence to specific protocols. This requirement restricts our ability to conduct extensive testing and analysis in regular laboratories. Lentivirus and vesicular stomatitis virus (VSV), which are normally used in neutralization assays and in screening for antiviral drugs, are based on the S protein of SARS‐CoV‐2.^20^, ^21^, ^22^ However, other major structural components affect the viral receptor binding properties.^23^ Additionally, an important issue with VSV‐derived pseudotyped viruses is the lingering presence of VSV remnants, leading to an elevated possibility of inaccurate positive outcomes.^24^ When preparing nonreplicative VSV pseudotyped viruses, it is important to remove any remaining G protein to prevent the occurrence of false‐positive results. To enhance the VSV‐based system, researchers have recently created potent, recombinant VSV–SARS‐CoV‐2 viruses that are highly contagious and capable of replication.^20^, ^25^ Nevertheless, no harmful capabilities of the VSV–SARS‐CoV‐2 virus have yet been completely examined, and the extensive manufacturing of these novel contagious particles may necessitate strict biocontainment settings. Furthermore, generating viral particles through numerous cycles of viral reproduction may result in the development of undesirable viral mutations and variations, required thorough examination and authentication.

To overcome the limitations of current pseudotyped viruses, a virus‐like particle (VLP) pseudotyped virus was constructed, containing the complete structural genes of SARS‐CoV‐2, encoding S, E, M, and N proteins,^26^ as well as several nonstructural protein genes. These nonstructural protein genes were combined with a reporter gene within a single expression plasmid. We constructed VLP pseudotyped viruses for different strains of SARS‐CoV‐2 using the methodology from Syed et al.^26^ We found that the titer of the VLP pseudotyped virus was relatively low for wild‐type SARS‐CoV‐2, and the omicron variant was unable to be packed. To improve the feasibility of this method, we further optimized the VLP pseudotyped virus. In this study, we optimized M and E proteins (M&E) complex, N protein, and nonstructural proteins to improve the titers of the SARS‐CoV‐2VLP pseudotyped viruses. We also screened several options to select the best S protein signal peptide. Based on this VLP pseudotyped virus system, we successfully developed a method to induce neutralizing antibodies directed against SARS‐CoV‐2. We also optimized various parameters, including the cell type, cell number, and viral inoculum. A comprehensive methodological verification process determined the specificity and reproducibility of the assay, and the correlation with the authentic virus. This pseudotyped virus effectively replicates the viral self‐assembly process, providing advantages for studying the infectivity and antigenicity of the virus. These advantages include excellent viral stability and a high degree of consistency with the authentic virus in terms of the neutralizing antibody titer. This research showcases the utility of SARS‐CoV‐2 VLP pseudotyped viruses for quickly quantifying neutralizing antibodies, viral mutations, and antiviral medications.

## 2 RESULTS

### 2.1 Construction and optimization of SARS‐CoV‐2 VLP pseudotyped viruses

**FIGURE 1:**
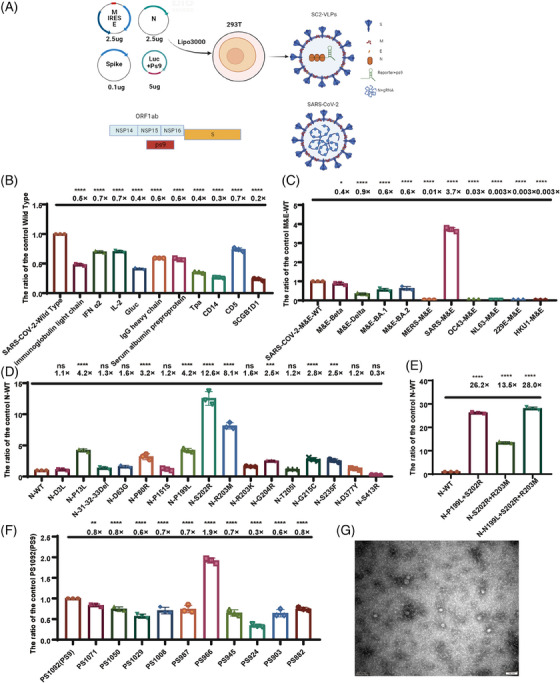
Construction and optimization of virus‐like particle (VLP) pseudotyped viruses. (A) Schematic representation of the construction of the VLP pseudotyped viruses. (B) Optimization of signal peptide for the S protein. Comparison of signal peptide of the severe acute respiratory syndrome coronavirus 2 (SARS‐CoV‐2) with 10 other signal peptides. (C) Optimization of M and E proteins for VLP pseudotyped viruses. Comparison of the effects of the M and E proteins of four SARS‐CoV‐2 variants (Beta, Delta, BA.1, and BA.2) and six other coronaviruses (MERS—CoV, SARS‐CoV‐1, OC43, NL63, 229E, and HKU1) on the viral titer. (D, E) Optimization of N protein for VLP pseudotyped viruses. Single or combined natural mutations were inserted into the N protein. (F) Optimization of nonstructural protein gene ORF1ab, PS9 (which contains a 1092‐bp nucleic acid fragment of nsp15–nsp16) was further truncated and compared. To statistically analyze multiple sets of data, one‐ or two‐way analysis of variance (ANOVA) tests and Dunnett's multiple comparisons test were utilized. The experimental data obtained from three repeated trials. The results are presented as means ± standard deviations (SD). Significance thresholds: *p < 0.05, **p < 0.01, ***p < 0.005, and ****p < 0.001.

The packaging of SARS‐CoV‐2 VLP pseudotyped viruses requires the cotransfection of four plasmids encoding structural proteins S, M, N, and E, and a packaging signal (PS9)‐containing reporter transcript (Figure 1A). The VLP pseudotyped viruses were achieved package with plasmids encoding wild‐type SARS‐CoV‐2 proteins that had low titers, but many omicron VLP pseudotyped viruses not successfully packaged. Therefore, we optimized the plasmids used to package the VLP pseudotyped viruses. The signal peptide plays a crucial role in both membrane targeting and membrane insertion, thereby potentially affects the encapsulation of pseudotyped viruses. Using a robust signal peptide derived from VSV‐G (VSVMEpv), the titer of the pseudotyped viruses was three times higher than the Japanese encephalitis virus (JEV) that was achieved with the weaker signal peptide (SPMEpv).^27^ To increase the package efficiency of the VLP pseudotyped viruses, we tested 10 types of signal peptides, including immunoglobulin light chain, interferon alpha 2 (IFN‐α2), interleukin 2 (IL2), *Gaussia* luciferase (Gluc), IgG heavy chain, serum albumin preproprotein, tissue plasminogen activator (TPA), CD14, CD5, and SCGB1D1. The results indicated that no other signal peptide increased the titers of the SARS‐COV‐2 VLP pseudotyped viruses, and the highest titer was still achieved with the signal peptide from SARS‐CoV‐2 itself (Figure 1B).

According to the relative studies,^30^ the particle number of VLP pseudotyped viruses is determined by the M&E protein. Therefore, we compared different M&E proteins found in SARS‐CoV‐2 mutants. Our analysis revealed no notable distinctions in the impacts of the M&E proteins between the SARS‐COV‐2 variants, encompassing Beta, Delta, BA.1, and BA.2. Moreover, the VLP pseudotyped viral titers decreased when we replaced the M&E protein with other coronaviruses that infect humans, such as MERS‐CoV, OC43, NL63, 229E, and HKU1. Interestingly, the titers of the VLP pseudotyped viruses increased 3.7‐fold when we replaced the M&E protein with SARS‐CoV‐1 (Figure 1C).

The protein N can bind to the transcription complex PS9, which carries the reporter gene. Therefore, we constructed VLP pseudotyped viruses with concern of natural mutation locations within the N proteins from the SARS‐CoV‐2 variants to identify mutation sites that potentially enhance the viral titer. By constructing VLP pseudotyped viruses with natural mutations in the N protein, we discovered that point mutations P13L, P80R, P199L, S202R, R203M, G204R, G215C, and S235F enhanced the titers of the VLP pseudotyped viruses (Figure 1D). Combining mutations P199L, S202R, and R203M within the serine–arginine (SR) linker region caused the most significantly improvement in titer. However, when we combined the P13L, P80R, and G204R mutant sites with the P199L, S202R, and R203M triple mutant, we observed no further increase in the viral titer (Figure S1). This indicates that the N protein had reached the maximum capacity in carrying nucleic acids. The combination of amino acid mutations P199L, S202R, and R203M in the SR linker region of the N protein increased the titers of the VLP pseudotyped viruses approximately 28‐fold (Figure 1E).

The ORF1ab gene within SARS‐CoV‐2 genome encodes nsp1–nsp16, which are crucial for the formation of the viral RNA polymerase.^28^ The PS9 plasmid, which contains a 1092‐bp nucleic acid fragment of nsp15–nsp16, plays a crucial role in the assembly of the VLP pseudotyped viruses, resulting in the highest viral titer.^26^ To further increase the efficiency of PS9, we conducted a truncation experiment on PS9, generating a total of 11 truncated packaging signals: PS882, PS903, PS924, PS945, PS966, PS987, PS1008, PS1029, PS1050, PS1071, and PS1092. Our results indicated that PS966 was better than PS9, and enhanced the titer of VLPs 1.9‐fold (Figure 1F).

We then combined the mutated N protein and the M&E protein of SARS‐CoV‐1 with the truncated PS966 packaging signal, the titers of the VLP pseudotyped viruses increased approximately 100‐fold.

Additionally, we also observe VLP pseudotyped viruses from a structural perspective. Due to the low yield of SARS‐CoV‐2 VLP pseudotyped viruses, we obtained negative staining electron microscopy images of the wild strain after concentration and purification and found that the diameter of SARS‐CoV‐2 VLP pseudotyped viruses particles was around 30 nm (Figure 1G). Compared with the normal SARS‐CoV‐2 virus particles with a diameter of 100 nm, the SARS‐CoV‐2 VLP pseudotyped virus particles shown a shorter diameter. The VLP pseudovirus was sphere in shape, inserted with spike proteins, similar to the SARS‐CoV‐2 particles.

### 2.2 Successfully packaging the omicron series SARS‐CoV‐2 VLP pseudotyped viruses and ensuring the stability of the SARS‐CoV‐2 VLP pseudotyped viruses

**FIGURE 2:**
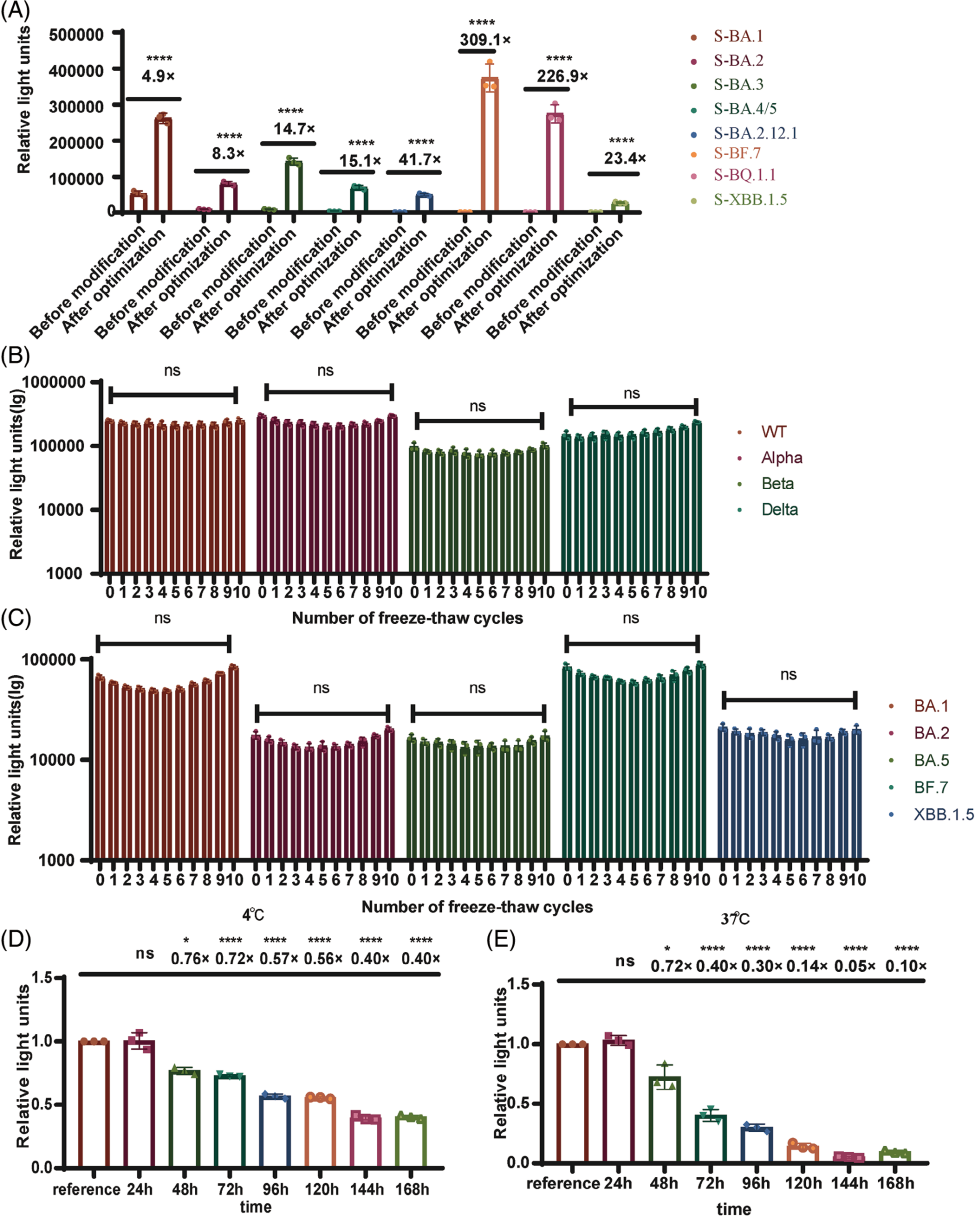
Comparison of the titers of omicron series virus‐like particle (VLP) pseudotyped viruses before and after optimization, and the stability of VLP pseudotyped viruses. (A) The optimized construct enhanced the titer of the omicron series VLP pseudotyped viruses. The luminescence values for the infection of VLP pseudotyped viruses were measured in 293T‐ACE2‐Furin cells before and after optimization. (B) Effect of repeated freeze–thaw on the titers of VLP pseudotyped viruses. Luminescence values were measured in 293T‐ACE2‐Furin cells after the wild‐type VLP pseudotyped viruses were freeze–thawed 10 times. (C, D) Effect of temperature on the titers of VLP pseudotyped viruses. Wild‐type VLP pseudotyped viruses were incubated at 4°C and 37°C for 7 days. Samples were collected once every 24 h and the titers of the VLP pseudotyped viruses measured in 293T‐ACE2‐Furin cells after different time intervals. To statistically analyze multiple sets of data, one‐ or two‐way analysis of variance (ANOVA) tests and Dunnett's multiple comparisons test were utilized. The experimental data obtained from three repeated trials. The results are presented as means ± standard deviations (SD). Significance thresholds: *p < 0.05, **p < 0.01, ***p < 0.005, and ****p < 0.001.

Using the intrinsic signal peptide from SARS‐CoV‐2, together with SARS‐CoV‐1 M&E, and the P199L, S202R, R203M triple mutants of the N protein, we successfully packaged all the omicron series VLP pseudotyped viruses that was unable to be packaged in the original system. The titers of the omicron series VLP pseudotyped viruses were all increased. The titer of BA.1 was enhanced 4.9‐fold, that of BA.2 8.3‐fold, BA.3 14.7‐fold, BA.4/5 15.1‐fold, BA.2.12.1 41.7‐fold, BF.7 309.1‐fold, BQ.1.1 226.9‐fold, and XBB.1.5 23.4‐fold (Figure 2A).

We also investigated the stability of the SARS‐CoV‐2 variants VLP pseudotyped viruses and the observation was taking place after 10 freeze–thaw cycles (Figure 2B,C), the titers of the VLP pseudotyped viruses remained relatively stable. When we stored the wild‐type VLP pseudotyped viruses at 4°C, they showed no significant changes in titer within 24 h. Only a fourfold reduction in titer was observed after 7 days (Figure 2D). Heat stability experiment was conducted at 37°C, the titers of the VLP pseudotyped viruses did not change significantly within 24 h, but decreased 1.36‐fold after 48 h (Figure 2E). Ensuring the stability of the VLP pseudotyped viruses offers economic advantages for virus transportation and experimental processes.

### 2.3 Optimization of neutralization assay based on SARS‐CoV‐2 VLP pseudotyped viruses

**FIGURE 3:**
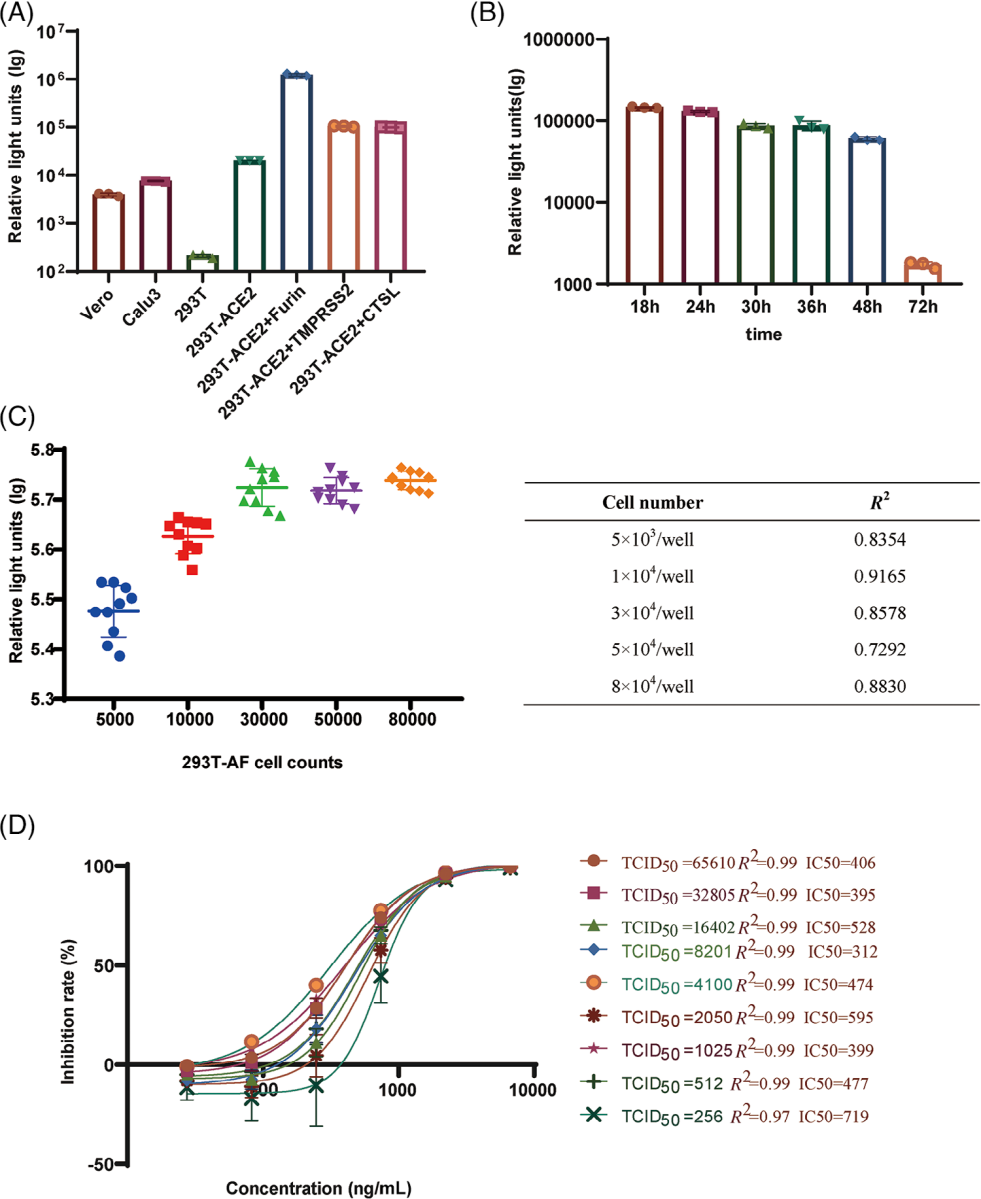
Optimization of severe acute respiratory syndrome coronavirus 2 (SARS‐CoV‐2) virus‐like particle (VLP)‐based pseudotyped‐virus‐based neutralizing assay (PBNA). (A) To select the best cell line for the assay, we tested seven different types of cells for their susceptibility to the SARS‐CoV‐2 VLP pseudotyped viruses. The *y*‐axis shows the absolute relative light units (RLUs) detected 18 h after VLP pseudotyped viral infection. (B) Selecting the appropriate time for neutralizing antibody testing. The *y*‐axis shows the absolute RLUs detected 18, 24, 30, 36, 48, and 72 h after VLP pseudotyped viral infection. (C) Optimization of cell numbers for neutralization. The *y*‐axis shows the absolute RLUs with different amounts of cells. The effect of cell number on neutralization was assessed by correlating cell numbers and their corresponding *R* ^2^ values. (D) Optimization of the viral dose for the SARS‐CoV‐2 VLP PBNA. To achieve effective neutralization of the SARS‐CoV‐2 VLP pseudotyped viruses, we optimized the viral dose for the assay. Various viral inocula in a dose range of 256–65,610 tissue culture infective doses (TCID_50_)/well were tested with the VLP PBNA method. To statistically analyze multiple sets of data, one‐ or two‐way analysis of variance (ANOVA) tests and Dunnett's multiple comparisons test were utilized. The experimental data obtained from three repeated trials. The results are presented as means ± standard deviations (SD). Significance thresholds: *p < 0.05, **p < 0.01, ***p < 0.005, and ****p < 0.001.

In order to identify specific cells that are responsive to neutralizing antibodies, infection trials were carried out on a variety of cell types, including Vero, Calu3, 293T, 293T‐ACE2‐Furin, 293T‐ACE2, 293T‐ACE2‐Cathepsin, and 293T‐ACE2‐TMPRSS2 cells. Among them, the cells expressing 293T‐ACE2‐Furin demonstrated the highest level of sensitivity (Figure 3A).

In order to establish the duration needed for neutralizing antibodies to be functional, we assessed the levels of luminescence at various time points following infection with VLP pseudotyped viruses, specifically at 18, 24, 30, 36, 48, and 72 h. It was observed that the viral titers of VLP pseudotyped viral titer viruses reached a peak and stayed constant from 18 to 24 h after infection (Figure 3B).

In order to determine the ideal quantity of 293T‐ACE2‐Furin cells for SARS‐CoV‐2 VLP pseudotyped viral infection, a titration of the SARS‐CoV‐2 VLP pseudotyped viruses was performed using varying cell numbers (5.00 × 10^3^–8.00 × 10^4^ per well). The highest viral titer was observed within a cell concentration range of 3.00 × 10^4^–8.00 × 10^4^ per well in the presence of a consistent amount of VLP pseudotyped viral preparation (Figure 3C). The correlation coefficients (*R* ^2^) achieved with cell inoculum ranging from 1.00 × 10^4^ to 3.00 × 10^4^ per well over 0.86, demonstrating a magnificent linear curve fitting. Nevertheless, when the input cell number exceeded 3.00 × 10^4^ per well, the *R* ^2^ values for the titration assay decreased dramatically. Therefore, we selected 3.00 × 10^4^ per well as the cell concentration for subsequent experiments.

We then optimized the viral inoculum by testing a dosage range of 256–65,610 median tissue culture infective doses (TCID_50_)/well for the SARS‐CoV‐2 pseudotyped‐virus‐based neutralizing assay (PBNA). Unexpectedly, the absolute half‐maximal inhibitory concentration (IC_50_) values shown no significant change, despite amounts of viral inoculum increased. In the viral titer range of 512–65,610 TCID_50_/well, there was no significant change in IC_50_, indicated by consistently high *R* ^2^ values of 0.99. However, when the viral titer decreased to 256 TCID_50_/well, a rapid increase in IC_50_ to 719 was observed, accompanied by a reduction in *R* ^2^ to 0.97 (Figure 3D). Furthermore, the IC_50_ was most sensitive at a viral titer of 2050 TCID_50_/well. Therefore, we defined that the optimal dose of viral inoculum was 2050 TCID_50_/well (Figure 3D).

### 2.4 Establishment and validation of the SARS‐CoV‐2 VLP PBNA

**FIGURE 4:**
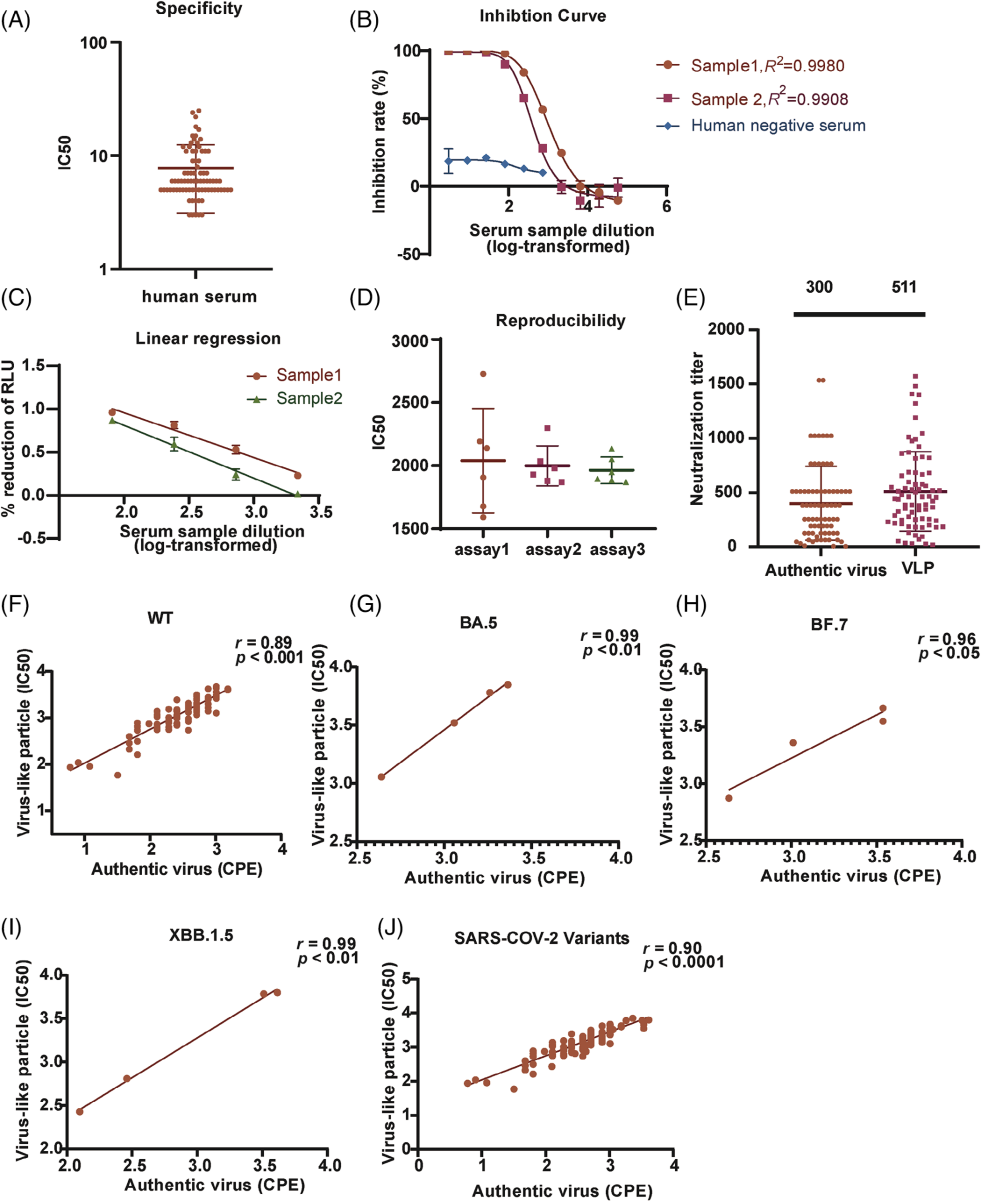
Validation of the severe acute respiratory syndrome coronavirus 2 (SARS‐CoV‐2) virus‐like particle (VLP) pseudotyped‐virus‐based neutralizing assay (PBNA) method. (A) Specificity of the VLP PBNA was assessed with a panel of 80 negative human samples. (B) Inhibition curves were plotted for the WHO International Standard for anti‐SARS‐CoV‐2 immunoglobulin and a negative human serum sample. These two samples showed typical four‐parameter inhibition curves when the log‐transformed dilution was plotted against the inhibition rate. The initial dilutions for the positive and negative samples were 1:10 and 1:3, respectively, followed by threefold serial dilution. (C) The linear range for the VLP PBNA occurred when the inhibition rate fell within a range of 1.6%–96%. In this range, there existed a direct and proportional relationship between the readout of the test sample and its dilution. (D) Reproducibility of the VLP PBNA was assessed with 18 tests on individual plates in three independent runs, using serum samples from two convalescent SARS‐CoV‐2 patients. Two convalescent serums also from the 72 serum samples were collected from Southern Medical University Nanfang Hospital, samples obtained from participants who were vaccinated with the anti‐SARS‐CoV‐2 vaccine. (E) Comparison of the geometric means of the VLP pseudotyped viruses and authentic virus. The geometric mean was calculated with separate tests on the neutralizing antibody titers of 72 serum samples using two methods. (F) Correlation analysis of the VLP PBNA and the authentic virus cytopathic effect (CPE) method. Correlation between VLP pseudotyped viruses and authentic virus was analyzed with the correlation function in the GraphPad Prism software. Statistical analysis was carried out using one‐way analysis of variance (ANOVA) and Dunnett's tests for multiple comparisons. Data from three independent experiments were collected.

The SARS‐CoV‐2 VLP PBNA was intended to be use in the analysis of human serum samples. To determine the specificity of this assay, a negative sample panel of 80 human sera was tested. The initial dilution was set to 1:3, followed by threefold serial dilution. The limit of detection (LOD) for the SARS‐CoV‐2 VLP PBNA was first determined. As demonstrated in Figure 4A, 60 serum samples displayed neutralizing antibody titers of <10, whereas the other 20 serum samples had neutralizing antibody titers of 10–25. The LOD was determined as the average titer of the negative samples plus 1.96 standard deviations (SD), resulting in a value of 16.9. Human serum samples were assigned cutoff values of 20 (Figure 4A). Using this threshold, the specificity for negative human sera was found to be 96.3%.

To determine the upper and lower limits of neutralizing antibody detection with the SARS‐CoV‐2 VLP PBNA, we diluted the WHO SARS‐CoV‐2 neutralizing antibody standard (1000 IU/mL) to onefold (Sample 1) and twofold (Sample 2). We then produced a series of dilutions in threefold increments, resulting in a total of 10 dilution levels in order to detect neutralizing antibodies. The results generated an S‐shaped curve, showing an inhibition rate over 98% at dilutions less than 81‐fold (>12.35 IU/mL). Conversely, the inhibition rate was <10% at dilutions greater than 2187‐fold (<0.457 IU/mL). Approximately 50% inhibition was observed near dilutions of 700‐fold (∼1.42 IU/mL). With a linear analysis, we determined the lower LOD for the S‐shaped curve as 0.15 IU/mL. The maximum inhibition rate of human negative serum was not exceeded 50% (Figure 4B).

The linear relationship between the signal and the concentration of a substance within a defined range is determined as linear range. To ascertain the linear range of the SARS‐CoV‐2 VLP PBNA, we tested the WHO International Standard against the SARS‐CoV‐2 VLP pseudotyped viruses. When comparing the log‐transformed dilution with the inhibition rate, typical inhibition curves with four parameters were observed for the positive samples in both cases (Figure 4B). In the case of positive examples, a linear correlation was observed between the dilution of the test sample and the readout showed an inhibition rate fell in 1.6%–96% range, with *R* ^2^ > 0.97 (Figure 4C).

To assess the reproducibility of the SARS‐CoV‐2 VLP PBNA, we combined serum samples from two patients who had recovered from SARS‐CoV‐2, and utilized them for testing the SARS‐CoV‐2 VLP pseudotyped viruses. A total of 18 tests of this particular sample were conducted on individual plates in three separate runs. The intra‐ and interassay coefficients of variation (CVs) averaged at 11.5% and 11.1%, respectively, meeting the acceptable criteria for a cell‐based assay (Figure 4D).

A correlation analysis of SARS‐CoV‐2 VLP PBNA and an assay based on the cytopathic effect (CPE) of the authentic virus were also conducted. A total of 72 convalescent serum samples were collected, and a correlation analysis was performed on the logarithmic values of authentic and VLP pseudotyped viral titers. The neutralizing antibodies directly against the authentic virus demonstrated a 300 geometric mean, whereas the mean of the neutralizing antibodies in the SARS‐CoV‐2 VLP PBNA method was 511 (Figure 4E), with a correlation coefficient of *r* = 0.89 (Figure 4F). Due to restrictions on obtaining authentic viruses, only three authentic viruses were obtained: BF.7, BA.5, and XBB.1.5. The correlation between VLP pseudotyped virus and authentic virus was examined, revealing a correlation coefficients of 0.99 for BA.5 (Figure 4G), 0.96 for BF.7 (Figure 4H), and 0.99 for XBB.1.5 (Figure 4I). The overall correlation coefficients between the wild strain and the three mutant strains of VLP pseudotyped virus and authentic virus were calculated to be 0.90 (Figure 4J).

Finally, we analyzed the precision and accuracy of the SARS‐CoV‐2 VLP PBNA. We tested three concentrations (high, 1000 U/mL; medium, 500 U/mL; low, 250 U/mL) of the SARS‐CoV‐2 neutralizing antibody national standard (first generation). The standard samples at each concentration were tested eight times separately, and the results showed a recovery rate ranging from 90.43% to 101.84%, indicating the impressive precision and accuracy of this method (Table S1).

Collectively, these findings suggested that the SARS‐CoV‐2 VLP PBNA established here is robust, and can be used to evaluate candidate vaccines and therapeutic drugs that target viral invasions.

### 2.5 Application of SARS‐CoV‐2 VLP PBNA

#### 2.5.1 Evaluation of cross reactivity in immunized guinea pig serum with VLP pseudotyped virus from 10 different variants

**FIGURE 5:**
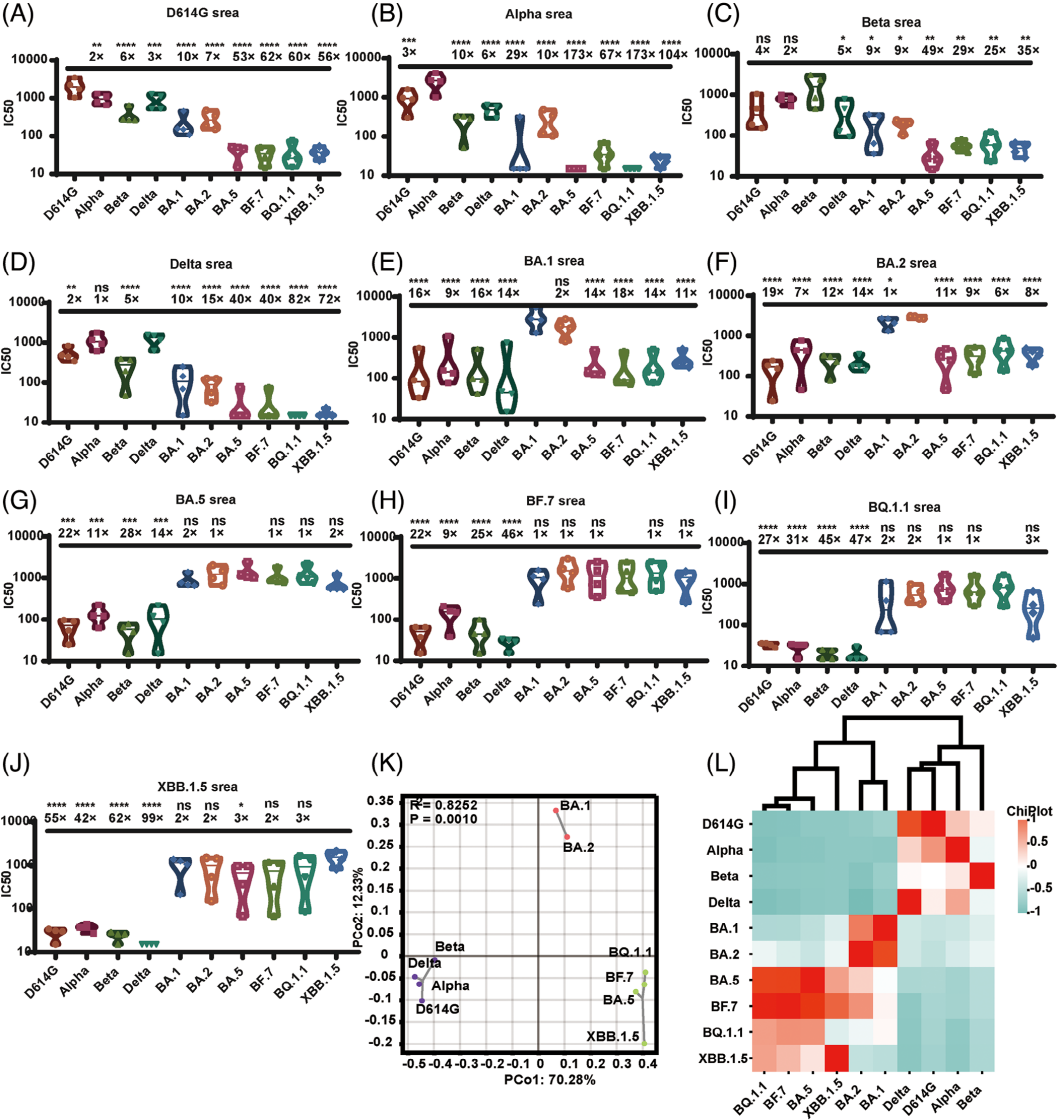
Analysis of cross neutralization and classification of sera from severe acute respiratory syndrome coronavirus 2 (SARS‐CoV‐2), generated by 10 spike proteins, against 10 different variants of SARS‐CoV‐2. Violin plots show the ID_50_ ratios of the virus‐like particle (VLP) pseudotyped viruses examined relative to the corresponding VLP pseudotyped viruses. The data shown are average values from three separate experimental trials. Data collected from serum samples obtained after the administration of three doses of (A) D614G, (B) Alpha, (C) Beta, (D) Delta, (E) BA.1, (F) BA.2, (G) BA.5, (H) BF.7, (I) BQ.1.1, or (G) XBB.1.5 are shown. (H) Categorization of spike protein variants of SARS‐CoV‐2 with principal components and correlation coefficient analyses. ID_50_ values for serum/virus pairs were log‐scale transformed, and vectors were constructed for each variant of SARS‐CoV‐2. (I) Heatmap shows a Spearman correlation coefficient (*R*) matrix for all immunogens. The correlation coefficient is represented by a scale bar. Red shows a positive correlation among virus variants neutralized by diverse sera, and blue indicates a negative correlation. Statistical analysis was performed with one‐way analysis of variance (ANOVA) and Dunnett's test for multiple comparisons. Significance of the difference between each group in a comparison with the homogeneous immunized group is indicated with asterisks. Data from three independent experiments were collected.

We acquired monovalent sera from immunizing guinea pigs with S protein of 10 SARS‐CoV‐2 mutant strains. We used these sera to detect the neutralizing antibody titers against corresponding 10 types of VLP pseudotyped viruses. To compare the differences in neutralizing antibodies between the mutant strains, the neutralizing antibody method we used had to be consistent. Our findings demonstrated that the neutralization effect against the homologous VLP pseudotyped viruses induced by all the sera was most effective when targeted to the same spike protein (Figure 5A–J).

When we used D614G, Delta Alpha, or Beta as the immunogen, the immune serum displayed effective neutralization activity against the aforementioned VLP pseudotyped viruses. However, there was notably limited cross‐neutralization activity against the Omicron variants.

When we used BA.1 or BA.2 as the immunogen, the serum raised showed lower (greater than sixfold lower) neutralization activity against different Omicron variants (BF.7, BA.5, BQ.1.1, and XBB.1.5). In particular, the serum generated by BA.1 showed a 14‐fold reduction in the neutralization of BA.5, an 18‐fold reduction in the neutralization of BF.7, a 14‐fold reduction in the neutralization of BQ.1.1, and an 11‐fold reduction in the neutralization of XBB.1.5.

The trio of immunogens, comprising BF.7, BA.5, BQ.1.1, and XBB.1.5, raised serum showing a greater than ninefold reduction in the ID_50_ values for D614G, Alpha, Beta, and Delta. Interestingly, this set of immunogens showed potent neutralizing effects against all investigated Omicron sublineage. In particular, BA.5, BF.7, and XBB.1.5 displayed a diverse array of neutralization potentials.

The immunogenicity relations of the SARS‐CoV‐2 variants were further examined with immunogen mapping. Antigenic spacing offered a concise overview of the antigenic connections among distinct immunogens, as shown in Figure 5K. The Spearman coefficients were computed for each immunogen, and a correlation matrix is presented as a heatmap in Figure 5L. The findings indicated that the 10 types of immunogens can be classified into three groups: group 1 (D614G, Alpha, Beta, and Delta), group 2 (BA.1 and BA.2), and group 3 (BA.5, BF.7, BQ.1.1, and XBB.1.5), consistent with the outcomes of neutralizing antibody of VSV system for pseudotyped viruses.^29^

#### 2.5.2 Neutralization activity of human convalescent serum against SARS‐CoV‐2 variant VLP pseudotyped viruses

**FIGURE 6:**
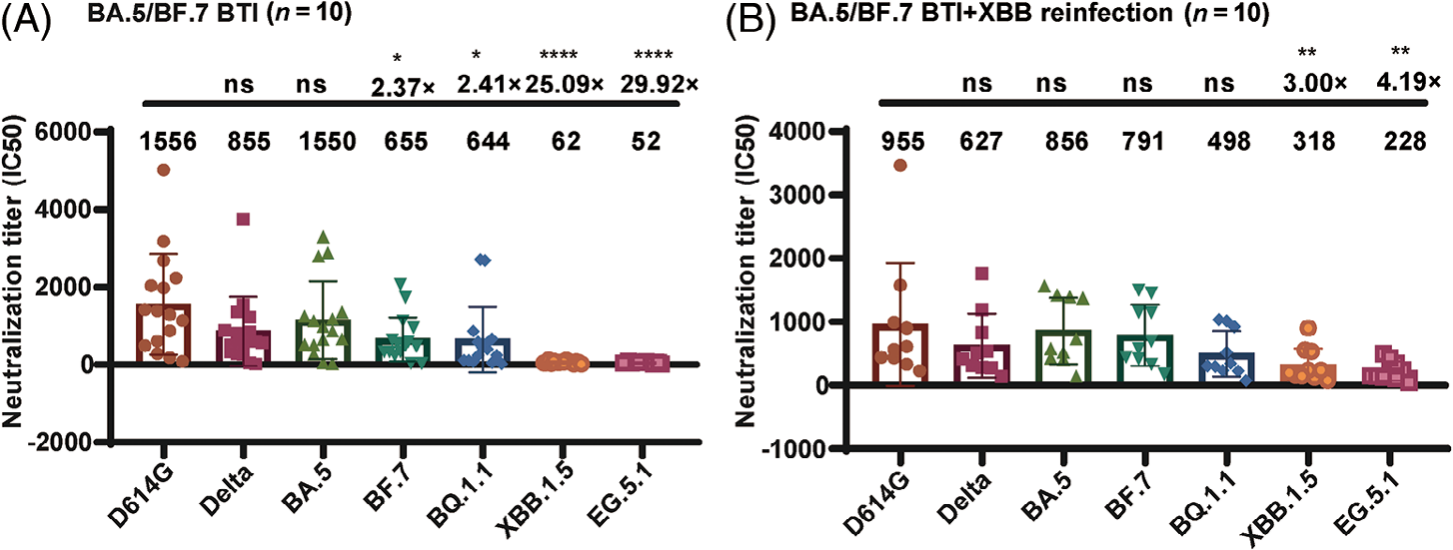
Humoral immune reaction after repeated Omicron infections in humans. (A) Neutralizing antibody titers against D614G, Delta, BA.5, BF.7, XBB.1.5, and EG.5.1 in convalescent sera from individuals after BA.5 or BF.7 breakthrough infections. Plasma antibody titers against virus‐like particle (VLP) pseudotyped virus D614G and variants were measured. (B) Neutralizing antibody titers against D614G, Delta, BA.5, BF.7, XBB.1.5, and EG.5.1 in convalescent sera from persons who experienced BA.5 or BF.7 breakthrough infections followed by reinfection with XBB. Plasma antibody titers against VLP pseudotyped virus D614G and variants were measured. Fold changes in titers against variants compared with that against D614G are shown above the line; geometric mean titers (GMTs) are shown. Statistical analysis was performed with one‐way analysis of variance (ANOVA) and Dunnett's test for multiple comparisons. Data from three independent experiments were collected. *n* refers to the number of individuals. Blood samples were collected 1–2 months after the last infection.

Plasma samples were collected from 16 convalescent individuals who had experienced breakthrough infections with BA.5 or BF.7, and 10 individuals who had experienced breakthrough infections with BA.5 or BF.7 followed by reinfection with XBB. All participants had received three doses of the CoronaVac vaccine prior to infection. Sera from the convalescent individuals infected with BA.5 or BF.7 showed strong neutralizing activity against the D614G, BA.5, Delta, BF.7, and BQ.1.1 strains. In contrast, these sera showed reduced neutralizing activity against the XBB.1.5 and EG.5.1 strains, with antibody titers decreasing 25‐ and 30‐fold, respectively (Figure 6A). Furthermore, sera from individuals with dual breakthrough infections displayed a broad‐spectrum neutralizing effect against strains D614G, BA.5, BF.7, Delta, BQ.1.1, XBB.1.5, and EG.5.1, and maintained their efficacy against strains XBB.1.5 and EG.5.1. Notably, the neutralizing antibody titers against strains XBB.1.5 and EG.5.1 only decreased three‐ and fourfold, respectively, demonstrating the production of broad‐spectrum neutralizing antibodies in individuals who experienced multiple Omicron breakthrough infections (Figure 6B).

## 3 DISCUSSION

Evaluating SARS‐CoV‐2 vaccines and therapeutic drugs currently involves using isolated authentic virus, which must be handled in a BSL 3 facility. Furthermore, authentic virus assay takes a minimum of 3 days to complete and is known to require a significant amount of manual labor. Simpler alternative methods can improve the advancement and assessment of vaccines and drugs, which aimed to blocking the invasion of SARS‐CoV‐2 into host cells.

PBNAs offer significant advantages over wild‐type‐virus‐based methods because they are both versatile and safer to handle. The versatility of pseudotyped viruses can be achieved by pseudotyping the virus with various outer membrane proteins or envelope proteins. This process effectively mimics the infection process of the authentic virus and provides the envelope proteins.^24^, ^30^ For example, both a VSV pseudotyped virus system and a human immunodeficiency virus (HIV) pseudotyped virus system have been generated in this manner. However, shortcomings still remained on the PBNAs. First, RNA viruses possess poor stability. Second, the exogenous membrane protein inserted into the VSV or HIV nuclear capsid is not as compatible as the own protein of the virus, which means that the quantity of inserted membrane protein may differ greatly from the number of protein molecules carried by the wild‐type virus. This difference is evident in the significantly higher titers of neutralizing antibodies measured compared with the authentic viruses.

In the present study, newly constructed SARS‐CoV‐2 VLP pseudotyped viruses were used to incorporated the SARS‐CoV‐2 structural proteins, and precisely reproduced the interactions between the proteins. Furthermore, the SARS‐CoV‐2 VLP pseudotyped viruses showed better consistency in their neutralizing antibody levels with the authentic virus. The pseudotyped virus also formed dense VLPs, which represent more stability than typical pseudotyped viruses. However, production yield of VLPs pseudotyped virus was not as high as the VSV and HIV pseudotyped virus systems. Akin to VSV pseudotyped virus, SARS‐CoV‐2 VLP pseudotyped viruses are also guaranteed in safe because the virus is essentially devoid of virulent viral components and involved in only a single round of replication. The VLP pseudotyped virus includes three additional structural proteins (M, E, and N) as well as the S protein. This pseudotyped virus can also be used for the in vitro screening of drugs directed against the M, E, and N proteins.

The neutralizing antibody detection method based on the SARS‐CoV‐2 VLP pseudotyped virus system has certain advantages over the SARS‐CoV‐2 VSV pseudotyped virus‐based system, but also shortcomings were still observed. Our research group has previously published a neutralizing antibody method for the VSV SARS‐CoV‐2 pseudotyped virus system.^21^ 293T cells stably express ACE2 and furin in the VLP system, whereas Huh7 and Vero are used as the sensitive cells in the VSV system, because VLP contained lower pseudotyped viral titer than VSV system, the receptor‐overexpressing cells must be used as sensitive cells. In terms of the detection time of the neutralizing antibody detection methods, the luminescence value of the VSV system peaked in 24 h and then began to decline, whereas the luminescence value of the VLP system slightly changed from 18 to 24 h, so luminescence able to be detected during this period, which shortened the detection time. In terms of the amount of virus added, the VSV system required 650 TCID_50_/well virus, whereas a wider range of virus can be added to the VLP system, with a relatively stable ID_50_ detection value. The cutoff value of the VSV system for the analysis of human serum is 30, whereas the cutoff value of the VLP system is 20, and the detection line is lower. The inhibition rate of SARS‐CoV‐2 constructed VSV system varied in a range from 20% to 80%, compared to the range from 1.6% to 96% of SARS‐CoV‐2 VLP system. Moreover, the dilution of the test sample correlates directly the resulting readout. VLP SARS‐CoV‐2 PBNA assay showed relatively low intra‐ and interassay CV, which were 11.5% and 11.1%, respectively. The PBNA assay for SARS‐CoV‐2 in the VSV system demonstrated consistent CV, with values of 15.9% within assays and 16.2% between assays. Furthermore, the detection of neutralizing antibodies with the VLP method correlates strongly with the detection of authentic virus (*r* = 0.9).

Protein M is a predominant protein found in the SARS‐CoV‐2 virus, with structural resembling akin to sugar transporter, it may play a role in the glycosylation of S protein and the interaction with antibodies.^31^ In addition, CoVs contain a glycosylated ectodomain at the N‐terminus of protein M that extends from the virus and binds to protein S, N, and E. Studies have shown that antibodies against protein M from SARS‐CoV collaborates with antibodies against proteins S and N to improve the process of neutralization.^32^, ^33^ This findings suggest that M potentially affects the neutralizing activities of anti‐S antibodies. At present, the mechanism behind the increased sensitivity of the VSV pseudotyped virus to antibody neutralization remain unsolved. One possibility is that this phenomenon might be linked to the reduced infectivity or stability of the VSV pseudotyped virus. Additionally, it is plausible that the VSV pseudotyped virus showed more accessible to be deactivated through antibody binding.

We also compared the VLP pseudotyped viruses with infectious SARS‐CoV‐2 and observed a strong direct correlation (*R* ^2^ = 0.9) in the amounts of induced antibodies, confirmed that the SARS‐CoV‐2 VLP pseudotyped virus can effectively substitute for SARS‐CoV‐2 in the measurement of neutralizing antibodies.

During our assessment of the neutralization assay, we discovered that the VLP PBNA technique exhibits exceptional sensitivity, precision, consistency, and resilience. In contrast to a traditional virus‐based assay, the VLP PBNA method is characterized by the objectivity and reduced labor intensity due to the use of a luminescent signal for data acquisition. In contrast, results of the authentic virus assay must be manually read by operators under a microscope. However, the ID_50_ of the SARS‐CoV‐2 VLP PBNA is basically consistent with the results of the authentic‐virus‐based assay.

However, this study also possesses limitations. Although the VLP pseudotyped virus contains all the structural proteins of the coronavirus, the genome packaged in the viral particle is only slightly over 2000 bp, which is far less than the entire 30‐kb genome of SARS‐CoV‐2, and the diameter of the viral particle is only around 30 nm. The yield of the VLP pseudotyped virus is lower than the SARS‐CoV‐2 pseudotyped virus in the VSV system, and the highest relative luminescence units (RLUs) are only in the hundreds of thousands. In addition, due to an unexpecting low titer of the SARS‐CoV‐2 VLP pseudotyped virus, animal model for in vivo testing has not been established, while previous studies claim that SARS‐CoV‐2 virus replicon particle system not only detects drugs and monoclonal antibodies in vitro, but also in vivo.^34^, ^35^, ^36^, ^37^, ^38^ In a relation analysis with authentic virus, 72 serum samples were selected for the comparison with wild strains, whereas only four samples were selected for the relation analysis of the variant strains with authentic virus, and only BA.5, BF.7, and XBB.1.5 were used as the variant strains. In the future, further study will analyze a large number of human sera and variant strains for methodological validation.

To summarize, the absence of fatal natural virus in this study is expected to significantly accelerate advancements in creating a vaccine and medications for SARS‐CoV‐2. Our aim is to provide these pseudotyped viruses of SARS‐CoV‐2 VLP, as well as the necessary guidelines, to individuals working on vaccines and medications for assessment of their potential products.

## 4 MATERIALS AND METHODS

### 4.1 Plasmids, cells, and serum

#### 4.1.1 Plasmids

Cloning plasmids encoding structural proteins: Plasmids encoding S, N, SARS‐M‐IRES‐E, and PS966 were generated on the pcDNA3.1 backbone. The NA sequences of E, M, and N were polymerase chain reaction (PCR) amplified from codon‐optimized plasmids and synthesized by General Biotech (Anhui) Co., Ltd. We used site‐directed mutagenesis to introduce mutations into the N protein.

#### 4.1.2 Cell lines

Maintained in Dulbecco's modified Eagle's medium (DMEM; HyClone) in a humidified incubator at 37°C with 5% CO_2_. 293T (CRL‐3216; American Type Culture Collection [ATCC]), Vero (ATCC, CCL‐81), Calu3 (ATCC, HTB‐55), 293T‐hACE2, 293T‐ACE2‐Furin, 293T‐ACE2‐TMPRSS2, and 293T‐ACE2‐CTSL cells are maintained in our laboratory.^39^

#### 4.1.3 Serum

The first WHO International Standard for anti‐SARS‐CoV‐2 immunoglobulin, coded 20/136, was purchased from National Institute for Biological Standards (NIBSC; UK Stem Cell Bank). The first national standard for anti‐SARS‐CoV‐2 immunoglobulin in China (lot 280034‐202001) is maintained in our laboratory. Eighty serum samples from Guangxi Province were collected in 2009–2010 and stored below −20°C by the National Institutes for Food and Drug Control. Two SARS‐CoV‐2‐positive human sera were donated by the Institute of Biological Products, National Institutes for Food and Drug Control. Seventy‐two Serum samples were collected from Southern Medical University Nanfang Hospital, samples obtained from participants who were vaccinated with the anti‐SARS‐CoV‐2 vaccine. Forty guinea pigs and serum obtained from our laboratory. Plasma samples were collected from 16 convalescent individuals who had experienced breakthrough infections with BA.5 or BF.7, and also from 10 individuals who had experienced breakthrough infections with BA.5 or BF.7 followed by reinfection with XBB.

### 4.2 Packaging and titration of VLP pseudotyped viruses

#### 4.2.1 Production of SARS‐CoV‐2 VLP pseudotyped virus

Plasmids coding for Cov2‐N (5.0 µg), CoV‐M‐IRES‐E (2.5 µg), CoV‐2‐Spike (0.5 µg), and Luc‐PS966 (5.0 µg) were mixed on a 25 cm dish in specified ratios to a total of 13 µg of DNA, then diluted in 250 µL of Opti‐MEM from Gibco. Next, 10 µL of P3000™ Reagent from Invitrogen was added to the mixed plasmids, followed by diluting 10 µL of Lipofectamine™ 3000 Transfection Reagent in 250 µL of Opti‐MEM and quickly combining it with the plasmid mixture for DNA complexation. The transfection mixture was left to incubate at 37°C for 20 min before being gently added to 293T cells in 5 mL of DMEM supplemented with fetal bovine serum and penicillin/streptomycin. Medium was refreshed at 12 h post‐transfection, and at 48 h post‐transfection, the VLP pseudotyped virus‐containing supernatant was harvested and filtered through a 0.45 µm syringe filter.

#### 4.2.2 Luciferase results

Fifty microliters of supernatant with the SARS‐CoV‐2 VLP pseudotyped virus was added to 50 µL of cell suspension (containing 30,000 receiver cells, 293T‐ACE2‐Furin) in each well of an opaque white 96‐well plate. The cells were allowed to attach and take up the VLP pseudotyped viruses overnight. After approximately 18 h, the culture medium was removed from the cell culture dish. Each well was added 100 µL of chemiluminescent substrate. The reaction was allowed to proceed for 2 min, and the luminescence signal was then measured with a luminometer.

### 4.3 Neutralization of VLP pseudotyped virus

Briefly, 100 µL samples were added to a 96‐well plate, diluted 1:10, and then serially diluted threefold. The 50 µL of VLP pseudotyped viruses (2050 TCID50) were mixed with the diluted samples in a 96‐well plate. The incubation of the mixture was carried out at 37°C for a duration of 1 h, mixed with 293T‐ACE2‐Furin cells (3 × 10^4^ cells/well), and then incubated at 37°C in a humidified atmosphere under 5% CO_2_. The chemiluminescent signals were measured in RLU after 18 h. IC_50_ was calculated with the Reed–Muench method.

### 4.4 Authentic virus neutralization assay

Authentic virus neutralization was performed as described below.^40^ First, 50 µL of serially diluted serums samples were added to 96‐well plates. Then, 50 µL of the authentic SARS‐CoV‐2 virus at a concentration of 2000 50% cell culture infective doses (CCID_50_)/mL was added. The plates were incubated at 37°C under 5% CO_2_ for 1 h. Vero cells (100 µL) at a concentration of 2 × 10^5^ cells/mL were added to each well of the plates. The plates were incubated at 37°C under 5% CO_2_ for a further 5 days. The CPE in each well was observed under a microscope by three different individuals. The IC_50_ was calculated with the Reed–Muench method. This assay was performed in a BSL 3 laboratory.

### 4.5 Observing the morphology of VLP pseudotyped viruses with negative staining EM

The collected SARS‐CoV‐2 VLP pseudotyped virus stock solution was concentrated with a 100‐kDa ultrafiltration tube (Millipore). The medium was then replaced with phosphate‐buffered saline, filtered and purified with a Capto™ Core 700 column (GE Healthcare Life Sciences). The resulting peak sample was then directly applied to a negative staining grid (freshly glow‐discharged 300‐mesh copper grid), incubated for 1 min, and stained with uranyl acetate for 2 min and 30 s. The resulting peak sample was imaged and negatively stained for electron microscopy (EM).

### 4.6 Statistical analysis

Data were analyzed with the GraphPad Prism 8.0 software (GraphPad). An unpaired two‐tailed Student's *t*‐test was employed to compare two sets of data. To statistically analyze multiple sets of data, one‐ or two‐way analysis of variance (ANOVA) tests and Dunnett's multiple comparisons test were utilized. The experimental data obtained from three repeated trials. The results are presented as means ± SD. Significance thresholds: *p < 0.05, **p < 0.01, ***p < 0.005, and ****p < 0.001.

## AUTHOR CONTRIBUTIONS

Youchun Wang, Weijin Huang, and Shuaiyao Lu revised the manuscript. Shuo Liu and Li Zhang wrote the manuscript and analyzed the experimental data. Shuo Liu, Li Zhang, Wangjun Fu, Ziteng Liang, Yuanling Yu, Tao Li, Jincheng Tong, Fan Liu, Jianhui Nie, and Qiong Lu performed the experiments. All authors have read and approved the final manuscript.

## CONFLICTS OF INTEREST STATEMENT

The authors declare no conflicts of interest.

## ETHICS STATEMENT

The research conducted at Nanfang Hospital of Southern Medical University (Guangzhou, China) was ethically approved (ethics approval number: ChiCTR2300078127). This study was approved by the Southern Medical University Nanfang Hospital (Guangzhou, China; ethics approval number: NFEC‐2023‐148). Human serum experiments were carried out in compliance with the Declaration of Helsinki, while the guinea pig experiments were authorized by the Institutional Animal Care and Use Committee at the National Institutes for Food and Drug Control (NIFDC). The guinea pig experiments adhered to the guidelines outlined in the Guide for the Care and Use of Laboratory Animals (Ethics number: Number 2020(B) 001).

## Supporting information

Supporting Information

## Data Availability

The data supported the results in this study are available from the corresponding author upon reasonable request.
