# Supplementary material for: Optimization and validation of a virus‐like particle pseudotyped virus neutralization assay for SARS‐CoV‐2
Source: MedComm (2020). 2024 Jun 14;5(6):e615. doi: 10.1002/mco2.615 (PMC11176738; doi:10.1002/mco2.615)
Supplement: Supplementary file 1 — Supporting Information [file MCO2-5-e615-s001.docx]

Optimization and validation of a virus-like particle pseudotyped virus neutralization assay for SARS-CoV-2

Shuo Liu^1,3^, Li Zhang^2^, Wangjun Fu^4,5^, Ziteng Liang^2,3^, Yuanling Yu^1^, Tao Li^2^, Jincheng Tong^2^ , Fan Liu^2^, Jianhui Nie^2^, Qiong Lu^2^, Shuaiyao Lu^6,*^, Weijin Huang^2,*^, Youchun Wang^1,3,*^

^1^Changping Laboratory, Beijing

^2^Division of HIV/AIDS and Sex-Transmitted Virus Vaccines, National Institutes for Food and Drug Control (NIFDC), Beijing, China

^3^Graduate School of Peking Union Medical College, Beijing, China

^4^CAS Key Laboratory of lnfection and lmmunity, National Laboratory of Macromolecules, lnstitute of Biophysics, Chinese Academy of Sciences, Beijing, China

^5^University of Chinese Academy of Sciences Beijing, China

^6^ Chinese Academy of Medical Sciences and Peking Union Medical College, Kunming, China


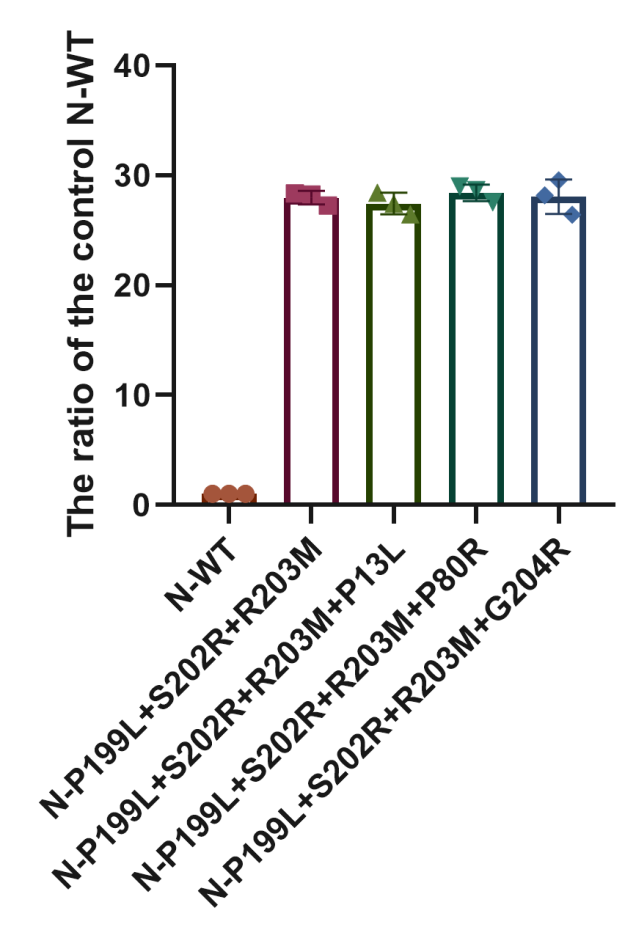


**Fig S1 The VLP pseudotyped viruses titer is not affected by the additional mutations of P13L, P80R, and G204R, based on the P199L, S202R, and R203M triple mutant.** **Infectivity analysis of SARS-CoV-2 N protein mutants. Chemiluminescence signals (in RLUs) were normalized against N-WT SARS-CoV-2 VLP pseudotyped viruses**

Table S1 The analysis of precision and accuracy of the detection of antiboy by VLP pseudotyped viruses

| Concentration（U/mL） | x ± s（U/mL） | CV% | Recovery（%） |
| --- | --- | --- | --- |
| 1000 | 904.29±89.17 | 9.86 | 90.43 |
| 500 | 497.90±57.68 | 11.59 | 99.58 |
| 250 | 254.61±24.06 | 9.45 | 101.84 |
